# Hypoxic pancreatic cancer derived exosomal miR-30b-5p promotes tumor angiogenesis by inhibiting GJA1 expression

**DOI:** 10.7150/ijbs.67675

**Published:** 2022-01-01

**Authors:** Kai Chen, Qi Wang, Xinxin Liu, Feng Wang, Yinmo Yang, Xiaodong Tian

**Affiliations:** 1 Department of General Surgery, Peking University First Hospital, Beijing, 100034, China.; 2 Department of Endoscopy Center, Peking University First Hospital, Beijing, 100034, China.

**Keywords:** Pancreatic cancer, ScRNA-seq, Angiognesis, Exosome, MiR-30b-5p

## Abstract

**Purpose:** Most patients with pancreatic ductal adenocarcinoma (PDAC) have vascular invasion and metastasis, leading to low surgical resection rate and dismal prognosis. Tumor angiogenesis is related to vascular invasion and metastasis. However, anti-angiogenesis therapeutic effects in PDAC are limited. Therefore, it is imperative to explore molecular mechanism of angiogenesis in PDAC.

**Experimental Design:** scRNA-seq data were utilized to delineatetranscriptional profiles of endothelial cells in PDAC. The in vitro and vivo angiogenesis models were used to explore the role of PDAC derived exosomes under hypoxic condition in tumor angiogenesis.

**Results:** Endothelial cells in PDAC had distinct gene expression profiles compared with normal pancreas. The marker genes of endothelial cells in PDAC were enriched for hypoxia and angiogenesis. MiR-30b-5p were significantly enriched in hypoxic PDAC cells derived exosomes, which could be transferred to HUVEC, resulting in the upregulation of miR-30b-5p. Hypoxic PDAC cells derived exosomes could promote tube formation and endothelial cells migration via miR-30b-5p mediated downregulation of gap junction protein GJA1. Moreover, hypoxic PDAC cells derived exosomes increased new microvascular density in vivo. Patients with PDAC had higher levels of total miR-30b-5p and exosomal miR-30b-5p in peripheral blood plasma than healthy subjects. In addition, there were significant correlations for the levels of total miR-30b-5p or exosomal miR-30b-5p between peripheral blood plasma and portal vein plasma.

**Conclusions:** Hypoxic PDAC cells derived exosomal miR-30b-5p promoted angiogenesis by inhibiting GJA1, and miR-30b-5p was a potential diagnostic marker for PDAC.

## Introduction

Pancreatic ductal adenocarcinoma (PDAC) has the highest level of malignancy among gastrointestinal tumors with the 5-year overall survival (OS) rate of only 9%^1^. The dismal prognosis of PDAC is mainly attributed to the lack of reliable early diagnostic markers, low surgical resection rate and chemoradiotherapy resistance. The majority of PDAC patients have lost the chance of operation at the time of diagnosis due to vascular invasion or distant metastasis^2^, ^3^. For PDAC patients who received radical surgery, half of them would exhibit recurrence and distant metastasis within 2 years after operation with the 5-year OS rate of 25-30%^4^. Tumor angiogenesis is closely related to tumor invasion and metastasis in PDAC. Despite recent progress in anti-angiogenesis therapy for PDAC, the overall treatment efficacy is limited^5^. Therefore, it is important to explore the mechanism of angiogenesis in PDAC in depth and develop new anti-angiogenesis strategies.

In recent years, the advent of single-cell sequencing technologies paved the way for revealing tumor heterogeneity comprehensively and identifying new cell subpopulations in PDAC. For example, Peng et al.^6^ analyzed 24 primary PDAC and 11 normal pancreatic tissues using single-cell RNA sequencing (scRNA-seq), and found two ductal cell subpopulations with different malignancy in PDAC, which indicated malignant ductal cells heterogeneity. Elyada et al.^7^ delineated cell atlases of human and mouse PDAC using scRNA-seq and demonstrated that cancer associated fibroblasts (CAFs) were divided into three distinct subpopulations: myofibroblastic CAFs (myCAFs), inflammatory CAFs (iCAFs), antigen-presenting CAFs (apCAFs). These findings reveal CAFs heterogeneity in PDAC and help develop novel target therapy strategies for certain types of CAFs. However, little is known about transcriptional profiles of endothelial cells in PDAC in single-cell resolution. The heterogeneity of endothelial cells in PDAC microenvironment remains unclear, which is an obstacle for exploring targeted anti-angiogenesis therapy strategies.

PDAC is more than insular masses of malignant ductal cells. Instead, they have sophisticated microenvironment composed of distinct cell populations participating in complex cell-to-cell communication to promote tumor growth together. Exosome is a kind of extracellular vesicle with a diameter of 40-160 nm (average, 100 nm), which has an endosomal origin^8^. Exosomes could encapsulate large amounts of bioactive molecules, such as DNAs, miRNAs, mRNAs, LncRNAs, proteins, lipids, metabolites^9^. A growing body of study revealed cell-to-cell communication mediated by exosomal miRNAs, involving pancreatic cancer cells, pancreatic stellate cells (PSCs), and immune cells^10^-^14^. Moreover, exosomes-based liquid biopsy technology has shown a wide application prospect for early diagnosis, tumor progress monitoring, prognostic prediction. For example, circulating exosomal Glypican-1 (GPC1), showed good specificity and sensitivity (AUC = 1.0) to distinguish healthy subjects, benign pancreatic diseases, and early and late stage of PDAC. Subsequently, multiple researches conducted experimental verification for the diagnostic value of exosomal GPC1^16^-^18^. But, cell-to-cell communication between endothelial cells and other compositions in PDAC microenvironment remains largely obscure.

In order to figure out the regulatory mechanism of angiogenesis in PDAC, single-cell RNA sequencing (scRNA-seq) datasets were acquired to delineate cell atlas of human PDAC. Here, we identified differential expression genes (DEGs) of endothelial cells between PDAC and normal pancreatic tissues, which were related to hypoxia and angiogenesis. Next, we utilized in vitro and in vivo angiogenesis models to examine the effects of exosomes secreted by tumor cells on endothelial cells. We found that hypoxic pancreatic cancer derived exosomes promoted angiogenesis through miR-30b-5p/GJA1 axis. In addition, total miR-30b-5p and exosomal miR-30b-5p in plasma were potential diagnostic markers to distinguish PDAC and health subjects.

## Results

### Single-cell sequencing delineated cell atlas of PDAC and normal pancreas

**Fig 1:**
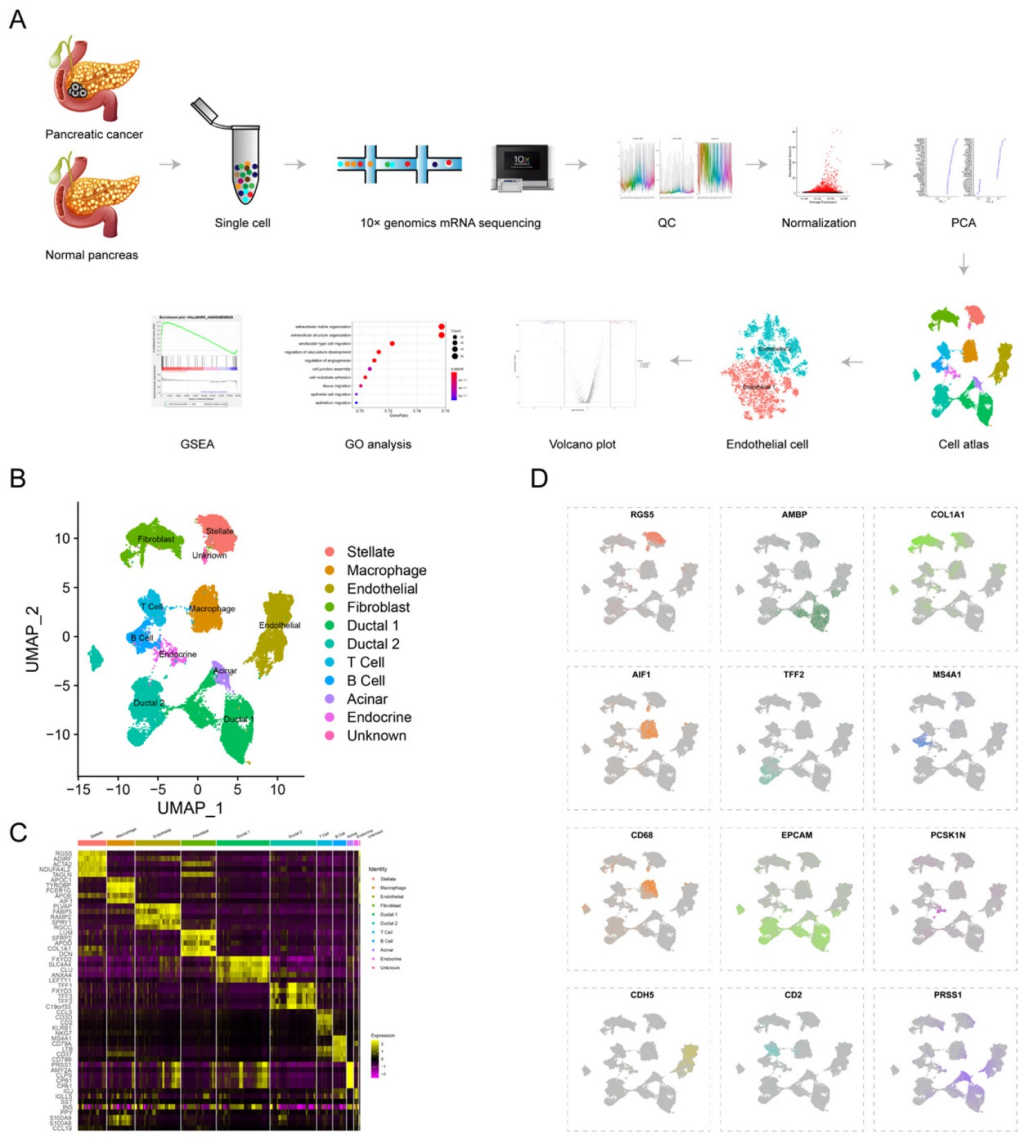
** scRNA-seq uncovers cell subpopulations in PDAC and normal pancreatic tissues.** (A) Workflow of scRNA-seq analysis. (B) u-MAP plot showing the cell subpopulations from 24 PDAC and 11 normal pancreatic specimens. (C) Dot plot showing top 5 marker genes across cell clusters. The bigger plot was, the higher proportion of cells expressing the marker gene was. The intensity of color represented the mean expression level of marker gene. (D) The normalized expression levels of signature genes of each known cell population were shown by u-MAP plot.

To reveal tumor heterogeneity in PDAC, a total of 24 PDAC (38201 cells) and 11 normal pancreases (14,838 cells) specimens were included to construct gene-cell matrix for scRNA-seq analysis (Supplementary Table S1). Flow of data processing was shown in Fig. 1A. Nine known cell clusters (Ductal, T cell, B cell, Stellate, Macrophage, Endothelial, Endocrine, Fibroblast and Acinar cell) were identified using Uniform manifold approximation and projection (u-MAP) (Fig. 1B and supplementary Fig. S1A-D). Marker genes of each cell cluster matched signature genes of cell populations reported in recent literatures (Fig. 1C-D, Supplementary Table S1)^6^, ^7^, ^19^, ^20^. Our previous study found that the proportions of immune cells and fibroblast cells were higher in PDAC compared with normal pancreas specimens, consistent with obvious desmoplasia and immune infiltration in PDAC (Supplementary Fig. S1E). The proportion of distinct cell populations varied among PDAC (T1-T24) and normal pancreas (N1-N11) specimens, suggesting the inter-patient heterogeneity (Supplementary Fig. S1F-G). In addition, we speculated the composition of cell cycle among different PDAC and normal pancreas specimens by CellCycleScoring and found PDAC had higher proportion of G2/M phase, indicating the active proliferation state (Supplementary Fig. S1H-J and L).

### Endothelial cells in PDAC were related to hypoxia and angiogenesis

**Fig 2:**
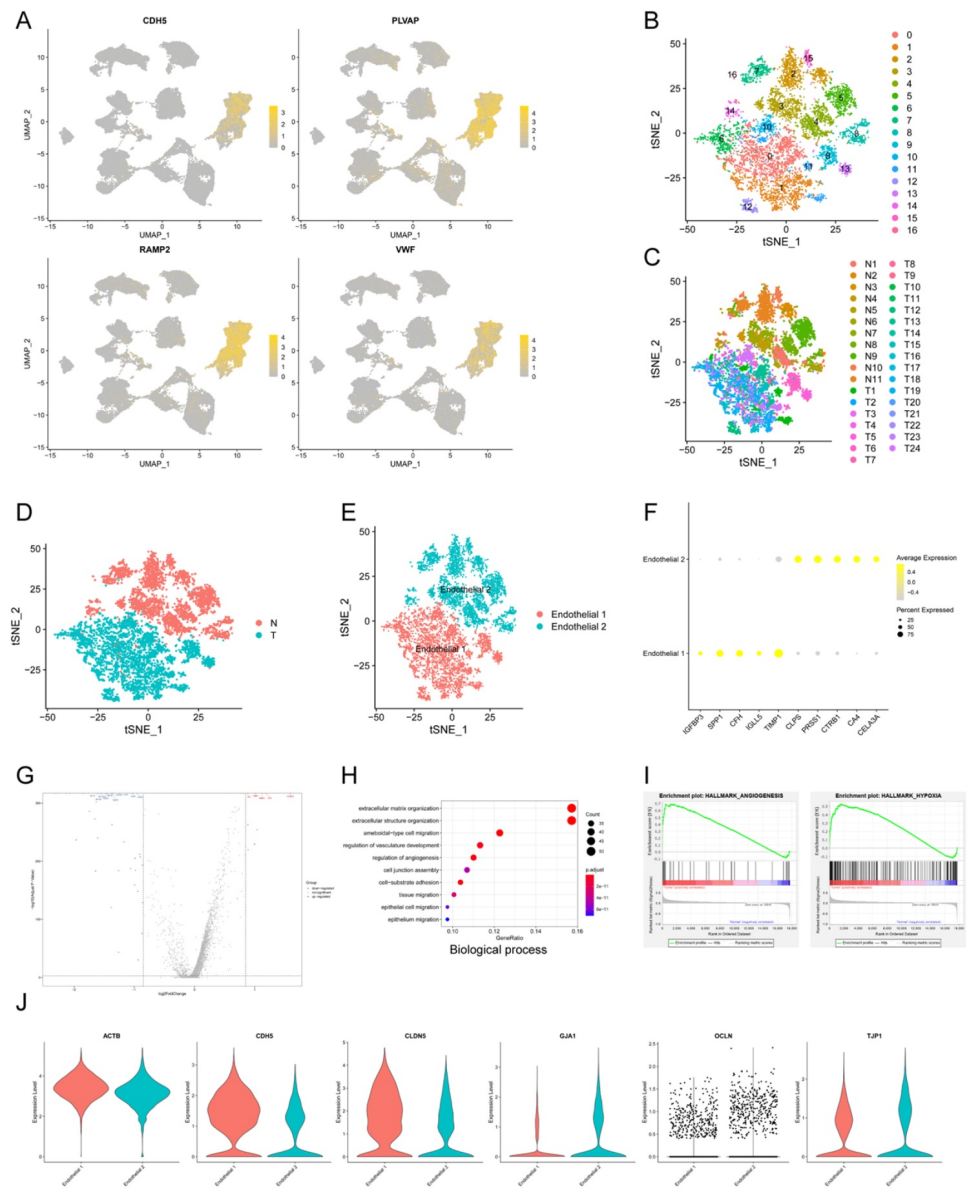
** The transcriptional profile characteristics of endothelial cells in PDAC.** (A) u-MAP plot showing the expression of signature genes of endothelial cells across cell clusters. The intensity of color represented expression level. (B-E) t-SNE plot showing original clusters (B), clusters from different specimens (C), clusters from PDAC or normal pancreatic specimens (D), named cell identifications (E). (F) Dot plot showing top 5 marker genes of two endothelial cell subpopulations. (G) Total differential expression genes between Endothelial 1 and Endothelial 2 were exhibited by volcano plot, red and blue dots represented the upregulated and downregulated genes respectively. (H) Gene ontology analysis showing biological process terms of upregulated genes in Endothelial 1, colorful dots represented the level of statistical significance. (I) Enrichment results of differential expression genes according to h.all.v7.2.symbols by GSEA analysis. (J) Violin plot showing the normalized expression level of cell junction proteins between Endothelial 1 and Endothelial 2.

Tumor angiogenesis plays a pivotal role in PDAC progression and metastasis^21^. In order to explore the expression pattern of endothelial cells in PDAC microenvironment, we isolated endothelial cells and conducted subclustering analysis using u-MAP method. Isolated cells expressed signature genes of endothelial cells, including CDH5, PLVAP, RAMP2, VWF, confirming their cell identification (Fig. 2A). In addition, we found new candidate makers of endothelial cells, including FABP5, SPRY1, RGCC, SDPR, CD320, SLC9A3R2. A total of 17 original clusters were identified (Fig. 2B). Clusters derived from PDAC and normal pancreas specimens were divided into two independent groups, Endothelial 1 and Endothelial 2 (Fig. 2C-E). There was significant difference between PDAC and normal pancreatic tissues for transcriptional profiles and cell cycle state of endothelial cells (Fig. 1K). Furthermore, Endothelial 1 had enriched expression of IGFBP3, SPP1, CFH, IGLL5, TIMP1, whereas Endothelial 2 specifically expressed CLPS, PRSS1, CTRB1, CA4, CELA3A (Fig. 2F).

We compared Endothelial 1 to Endothelial 2, and found 338 up-regulated genes and 251 down-regulated genes (q-value < 0.05, logfc > 0.25) (Fig. 2G). Endothelial 1 representing PDAC had higher expression level of HIF1A compared with Endothelial 2 representing normal pancreatic tissues, suggesting hypoxia and low microvascular density as hallmarks of PDAC. GO analysis showed that upregulated genes in Endothelial 1 were enriched for extracellular matrix organization, regulation of vasculature development, regulation of angiogenesis, cell junction assembly and epithelial cell migration (Biological process); extracellular matrix structural constituent, growth/insulin-like growth factor binding (Molecular function) (Fig. 2H and Supplementary Fig. S2A). KEGG and DO analysis indicated that upregulated genes in Endothelial 1 were related to PI3K-Akt signaling pathway, ECM-receptor interaction and malignant diseases (Supplementary Fig. S2B-C). In addition, we performed the annotation of DEGs by GSEA tool, and found that upregulated genes in Endothelial 1 were enriched for HALLMARK_ANGIOGENESIS (NES = 2.382) and HALLMARK_HYPOXIA (NES = 2.397) (Fig. 2I and Supplementary Fig. S2D).

Multiple studies demonstrated that cell junction proteins, such as TJP1 and CDH5, regulated tumor angiogenesis to promote tumor invasion and metastasis^22^-^24^. Next, we detected expression levels of various cell junction proteins in PDAC and normal pancreatic tissues using scRNA-seq data. Endothelial cells in PDAC had higher expression of CDH5 and CLDN5, whereas lower expression of GJA1, OCLN and TJP1 (Fig. 2J). These results suggested that dysregulated cell junction proteins might contribute to tumor angiogenesis in PDAC.

### Hypoxic pancreatic cancer cells derived exosomes promoted tube formation and endothelial cell migration

**Fig 3:**
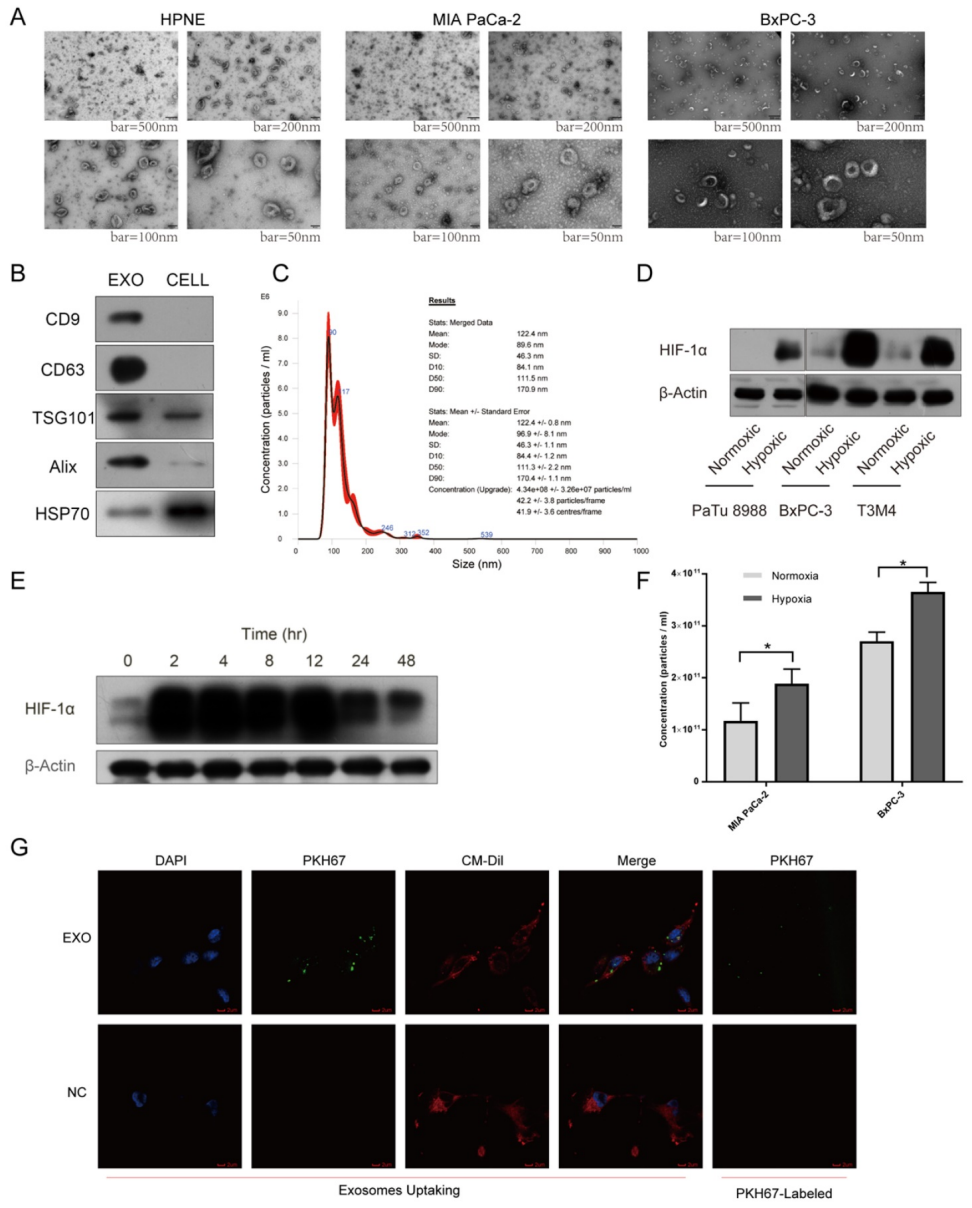
** Exosomes identification and hypoxic incubator chamber validation.** (A) Electron microscopy images of exosomes from various cell lines. Typical saucer-like exosomes with a range of 30 - 160 nm in diameter were detected, bar = 50 nm, 100 nm, 200 nm and 500 nm. (B) Isolated exosomes expressed maker proteins of exosomes compared with parental cells. (C) Representative NTA image showing the distribution of the size and concentration of isolated exosomes. (D) The expression of HIF-1α in pancreatic cancer cells under hypoxic and normoxic conditions. (E) The expression levels of HIF-1α at different times under hypoxic condition. (F) The concentration of exosomes secreted by pancreatic cancer cells under hypoxic and normoxic conditions was calculated by NTA. (G) Exosomes uptake, exosomes were labeled with PKH67 (Green), HUVEC was stained with DAPI (Blue) and CM-Dil (Red). The top right image showing that PKH67-exosomes were green spherical particles in a nanoscale.

A growing body of evidence supported that exosomal miRNAs could mediate cell-to-cell communication among different compositions of tumor microenvironment, promoting tumor proliferation and metastasis together^25^. To explore cell-to-cell communication between tumor cells and endothelial cells mediated by exosomes, we first utilized incubator chamber flushed with 5% CO2 and 95% N2 to simulate hypoxic microenvironment in PDAC, then harvested supernatants of cells with multilayer culture flask in different oxygen conditions (Supplementary Fig. S2E-F). Isolated vesicles were characterized and quantified using transmission electron microscopy (TEM), immunoblot and nanoparticle tracking analysis (NTA). Typical saucer-like vesicles with a range of 30-160 nm in diameter were observed, which were extracted from different cell lines supernatant (Fig. 3A). Exosomal markers, including CD9, CD63, TSG101, Alix, HSP70, were detected in these vesicles (Fig. 3B). NTA results showed that the size of isolated vesicles was about 120nm (Fig. 3C). Next, we named these vesicles exosomes. Multiple cell lines expressed higher levels of HIF-1α under hypoxic condition than normoxic condition, verifying the reliability of hypoxic incubator chamber (Fig. 3D). Cultured cells maintained high level of HIF-1α after 48 hours under hypoxic condition, thus we chose 48 hours as the time to harvest cell supernatant to extract exosomes. Furthermore, we found MIA PaCa-2 and BxPC-3 secreted higher concentration of exosomes under hypoxia than normoxia with the same number of cells (Fig. 3F). After co-incubation with exosomes labeled with PKH67 dye for 4 hours, HUVEC exhibited an efficient uptake of labeled exosomes (Fig. 3G).

**Fig 4:**
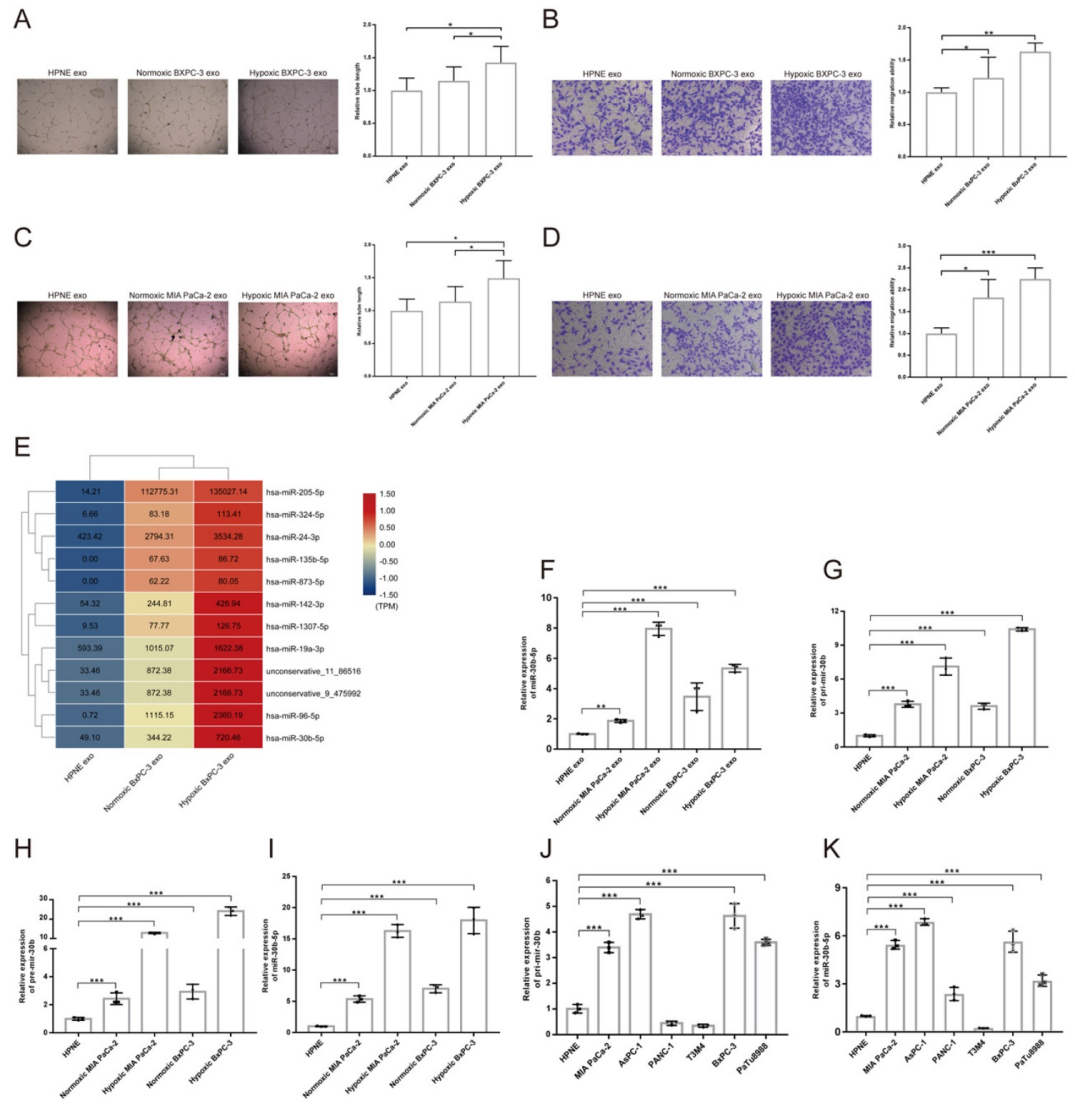
** Pancreatic cancer cells derived exosomes promoted angiogenesis and exosomal miRNAs identification.** (A-D) Representative images and statistical results of tube formation and endothelial cells migration for BxPC-3 (A-B), MIA PaCa-2 (C-D). (E) Heatmap sowing the expression of candidate miRNAs according to exosomal small RNA-seq, the original expression levels of miRNAs were shown. (F) RT-qPCR showing the relative expression level of miRNAs in different exosomes. (G-I) RT-qPCR showing the relative expression level of Pri-mir-30b (G), Pre-mir-30b (H), miR-30b-5p (I). (J-K) The expression level of Pri-mir-30b and miR-30b-5p in multiple parental cell lines.

To investigate the effect of hypoxic pancreatic cancer cells derived exosomes on angiogenesis, we performed tube formation and migration assay. The results showed that exosomes extracted from hypoxic MIA PaCa-2 (Hp_MIA PaCa-2_Exos) and BxPC-3 (Hp_BxPC-3_Exos) increased total tube formation length and endothelial cells migration compared with normoxic MIA PaCa-2 (No_MIA PaCa-2_Exos) and BxPC-3 (No_BxPC-3_Exos) and normoxic HPNE (No_HPNE_Exos) (Fig. 4A-D). However, we did not find Hp_MIA PaCa-2_Exos and Hp_BxPC-3_Exos promoted vascular permeability and transendothelial migration (Supplementary Fig. S3A-B).

### MiR-30b-5p was enriched in hypoxic pancreatic cancer cells derived exosomes

Exosomes regulated cell-to-cell communication through transferring proteins, mRNAs, miRNAs, LncRNAs, lipids and metabolites to recipient cells^26^. Exosomal small RNA-seq was designed to screen candidate miRNAs in hypoxic pancreatic cancer cells derived exosomes, that promoted tumor angiogenesis. We searched for differential expression miRNAs among No_HPNE_Exos, No_BxPC-3_Exos and Hp_BxPC-3_Exos (Supplementary Fig. S3C-F). Total 12 miRNAs were highly expressed in Hp_BxPC-3_Exos (Fig. 4E). RT-qPCR results showed that only miR-30b-5p was highly expressed in both Hp_BxPC-3_Exos and Hp_MIA PaCa-2_Exos (Fig. 4F). In addition, precursors (Pri-mir-30b, Pre-mir-30b) and mature miR-30b (miR-30b-5p) were specifically enriched in hypoxic parental cells (Fig. 4G-I). We also examined the expression level of Pri-mir-30b and miR-30b-5p in other cells. Besides MIA PaCa-2 and BxPC-3, AsPC-1, PANC-1 and PaTu8988 expressed high level of miR-30b-5p (Fig. 4J-K). Thus, we selected miR-30b-5p as the candidate for further investigation.

### Hypoxic pancreatic cancer cells derived exosomes promoted angiogenesis by transferring miR-30b-5p to endothelial cells

**Fig 5:**
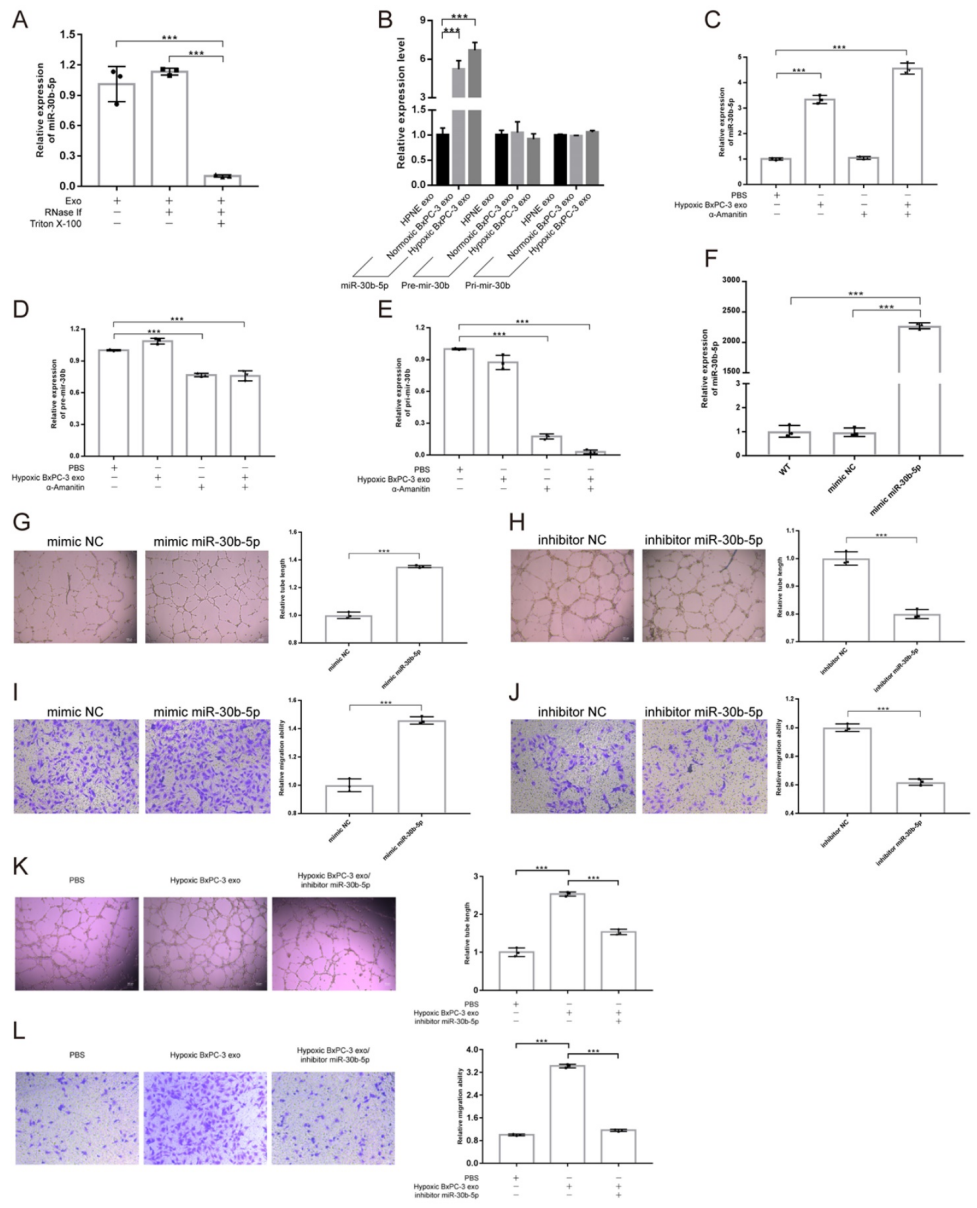
** Hypoxic pancreatic cancer cells derived exosomal miR-30b-5p promoted angiogenesis.** (A) Exosomal miR-30b-5p protection assay, the expression of exosomal miR-30b-5p were detected with or without RNase if and Triton X-100 treatment. (B) RT-qPCR detected the relative expression of Pri-mir-30b, Pre-mir-30b and miR-30b-5p after exosomes treatment for 24 hours. (C-E) RT-qPCR detected the relative expression of Pri-mir-30b (C), Pre-mir-30b (D), and miR-30b-5p (E) after the co-incubation of exosomes and RNA polymerase II inhibitor (α-Amanitin) in HUVEC. (F) The expression of miR-30b-5p in HUVEC after being transfected with miR-30b-5p mimic or scrambled control. (G-J) Representative images and statistical results of tube formation and endothelial cells migration for miR-30b-5p mimic (G, I) and inhibitor (H, J). (K-L) Rescue experiments of tube formation (K) and endothelial cells migration (L). The co-incubation of hypoxic BxPC-3 derived exosomes and miR-30b-5p inhibitor in HUVEC.

Exosomal miRNAs protection assay showed that the concentration of exosomal miR-30b-5p was not influenced by RNase, but decreased dramatically after additional Triton X-100 treatment, indicating that exosomes could protect miR-30b-5p from RNase degradation (Fig. 5A). Compared with No_HPNE_Exos and No_BxPC-3_Exos, HUVEC significantly upregulated the expression level of miR-30b-5p after co-incubation with Hp_BxPC-3_Exos for 24 hours, but Pri-mir-30b and Pre-mir-30b remained unchanged (Fig. 5B). Furthermore, HUVEC upregulated miR-30b-5p by internalizing Hp_BxPC-3_Exos even in the presence of RNA polymerase II inhibitor α-Amanitin (Fig. 5C). However, the expression of Pri-mir-30b and Pre-mir-30b in HUVEC were suppressed by α-Amanitin (Fig. 5D-E). Therefore, HUVEC upregulated the expression of miR-30b-5p via internalizing exosome-encapsulated mature miR-30b-5p, but not de novo synthesis after exosomes treatment.

In order to investigate the role of miR-30b-5p in angiogenesis, we transfected HUVEC with miR-30b-5p mimic. After transfection, HUVEC significantly upregulated the expression level of miR-30b-5p (Fig. 5F). Overexpression of miR-30b-5p significantly increased total tube formation length and endothelial cells migration (Fig. 5G, I). Inhibition of miR-30b-5p had the opposite effect (Fig. 5H, J). Moreover, rescue experiments showed that the inhibition of miR-30b-5p partially reduced the ability of Hp_BxPC-3_Exos to promote tube formation and endothelial cells migration (Fig. 5K-L). Collectedly, these data suggested that hypoxic pancreatic cancer cells derived exosomes may promote angiogenesis via exosome-encapsulated miR-30b-5p.

### MiR-30b-5p directly regulated GJA1

**Fig 6:**
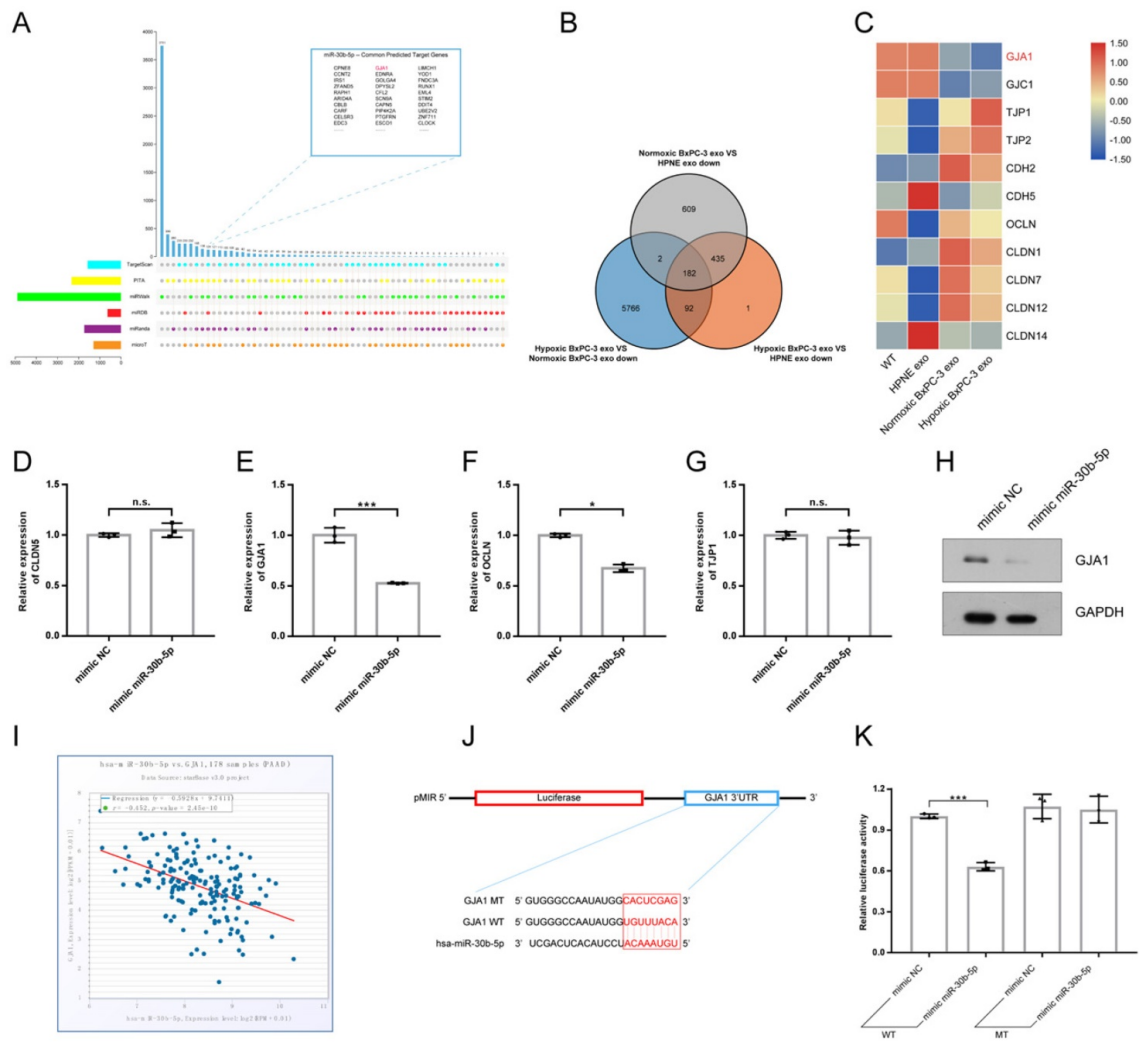
** MiR-30b-5p directly regulated GJA1.** (A) The prediction of target genes of miR-30b-5p using multiple miRNA databases. Upset plot showing the common target genes in top right box. (B) Venn plot showing the common downregulated genes in HUVEC after exosomes treatment. (C) Heatmap showing the expression of cell junction proteins according to RNA-seq. Red box and green box represented high expression and low expression respectively. (D-G) RT-qPCR verified the expression of selected cell junction proteins in HUVEC after the overexpression of miR-30b-5p. (H) Western blotting verified the expression of GJA1 after the overexpression of miR-30b-5p. (I) The correlation analysis between has-miR-30b-5p and GJA1 based on TCGA_PAAD cohort. (J) The dual-luciferase reporter vector design for the 3' UTR of GJA1. Red box indicated seed region of miR-30b-5p. (K) Relative luciferase activities across groups were tested in dual-luciferase reporter assay.

Multiple miRNA databases were used to predict target genes of miR-30b-5p. Total of 124 common target genes were identified using different prediction algorithm (Fig. 6A). Then, RNA-seq was performed to further screen target genes of exosomal miR-30b-5p (Supplementary Fig. S3G). Compared with No_HPNE_Exos, 617 genes were downregulated in HUVEC in common after treatment with No_BxPC-3_Exos or Hp_BxPC-3_Exos, and 182 genes were the least expressed in Hp_BxPC-3_Exos group (Fig. 6B). The expression levels of cell junction proteins among different groups were shown in the heatmap (Fig. 6C). Interestingly, HUVEC had the lowest expression level of GJA1 after Hp_BxPC-3_Exos treatment. Next, we aimed to validate whether miR-30b-5p could regulate GJA1 at post-transcriptional level. RT-qPCR and Western blot results showed that overexpression of miR-30b-5p significantly downregulated the expression of GJA1 (Fig. 6D-H). There was a negative correlation between has-miR-30b and GJA1 in TCGA_PAAD cohort (r = -0.452, P_value = 2.45e-10). Dual-luciferase reporter assay was conducted to further validate that miR-30b-5p could directly suppress the expression of GJA1 by combining with 3'UTR region. Results showed that overexpression of miR-30b-5p significantly decreased the activity of luciferase, whereas this effect was abolished after the mutation of binding region (Fig. 6J-K).

### Hypoxic pancreatic cancer cells derived exosomes promoted angiogenesis via miR-30b-5p/GJA1 axis

**Fig 7:**
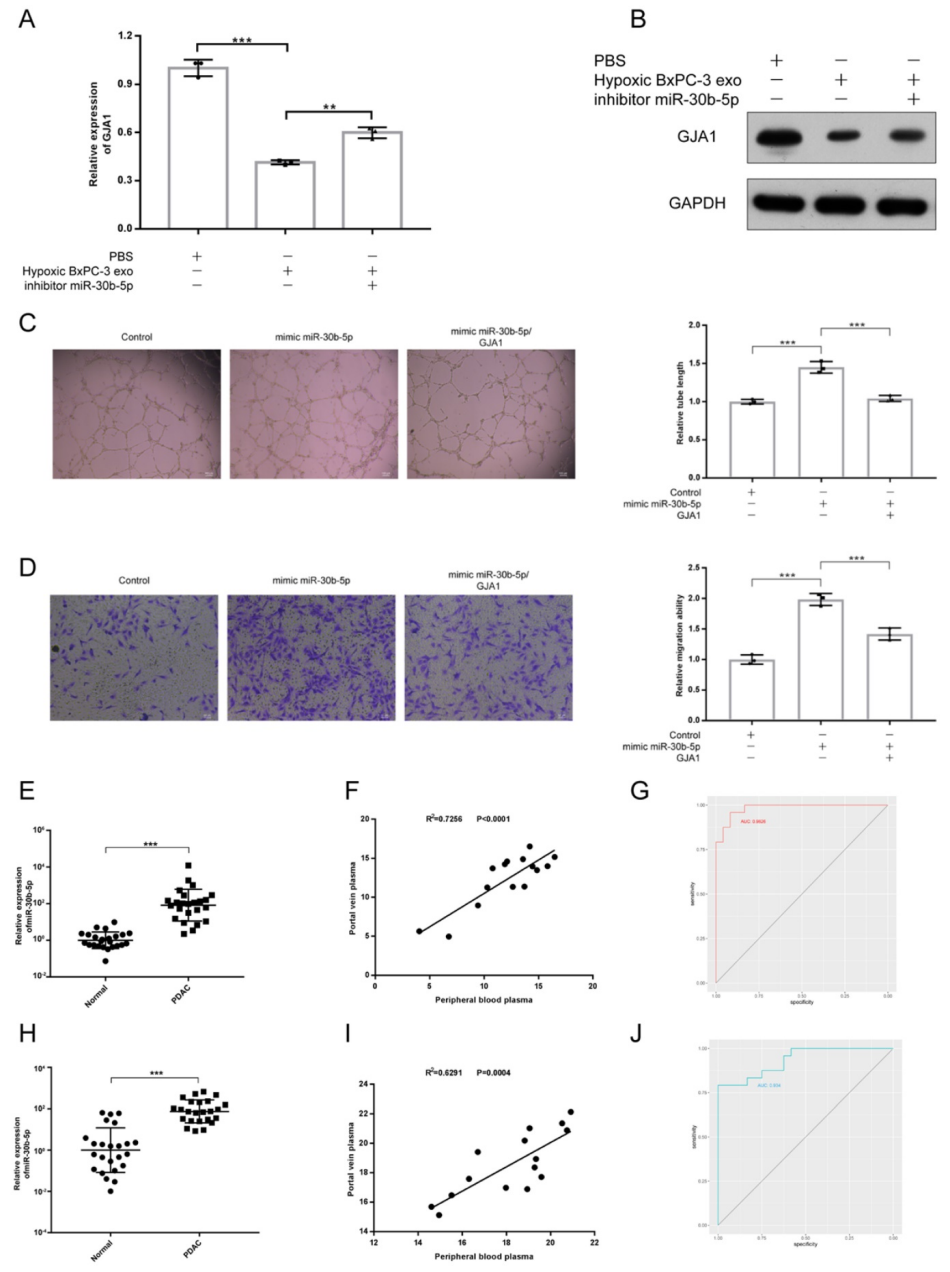
** Exosomal miR-30b-5p promoted angiogenesis through suppressing GJA1.** (A-B) Rescue experiments, the expression of GJA1 in RNA (A) and protein (B) level was detected after co-incubation of hypoxic BxPC-3 derived exosomes and miR-30b-5p inhibitor. (C-D) Rescue experiments, the tube formation and endothelial cell migration were tested after co-transfection of miR-30b-5p mimic and overexpression vector of GJA1. (E) The relative expression of total miR-30b-5p in plasma between PDAC and healthy subjects. (F) The correlation for expression of total miR-30b-5p between peripheral blood plasma and portal vein plasma. (G) ROC plot showing the diagnostic value of total miR-30b-5p in plasma to distinguish PDAC and healthy subjects. (H) The relative expression of exosomal miR-30b-5p in plasma between PDAC and healthy subjects. (I) The correlation for expression of exosomal miR-30b-5p between peripheral blood plasma and portal vein plasma. (J) ROC plot showing the diagnostic value of exosomal miR-30b-5p in plasma to distinguish PDAC and healthy subjects.

To validate whether PDAC promoted angiogenesis by transferring exosomal miR-30b-5p to endothelial cells to suppress the expression of GJA1, rescue experiment was designed. We found that the downregulation of GJA1 at RNA and protein levels by Hp_BxPC-3_Exos could be rescued by the inhibition of miR-30b-5p (Fig. 7A-B). Furthermore, overexpression of GJA1 partially decreased pro-angiogenesis of miR-30b-5p (Fig. 7C-D).

### Hypoxic pancreatic cancer cells derived exosomes promoted angiogenesis in vivo

**Fig 8:**
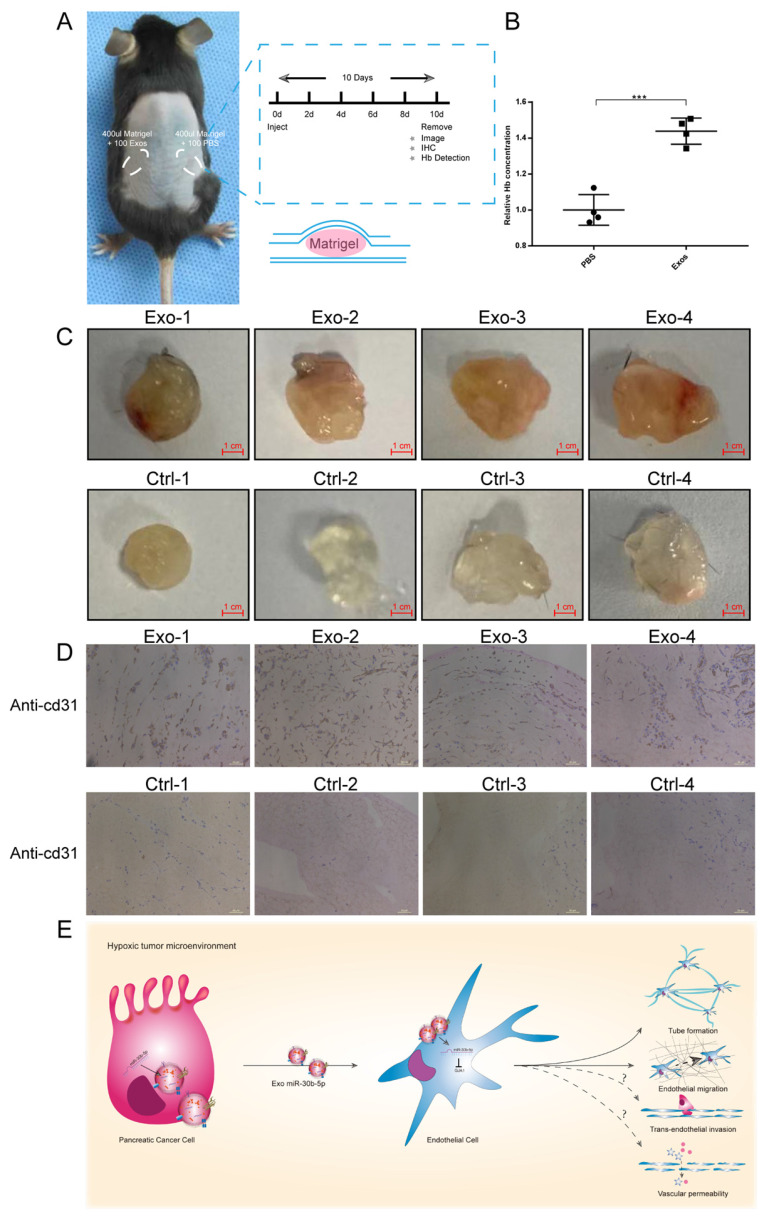
** Hypoxic exosomal miR-30b-5p promoted angiogenesis in vivo.** (A) Graphic scheme describing the experimental workflow of matrigel plug assay. (B) Relative Hb concentration of each matrigel plug was tested. (C) Representative images of matrigel plugs. (D) IHC results for each formalin-fixed matrigel plug section for cd31. (E) Illustrated summary of hypoxic pancreatic cancer cells derived exosomes promoting angiogenesis. Dotted line indicated incomplete results.

To evaluate the function of exosomes secreted by cancer cells in vivo, we conducted matrigel plug assay. Equal amounts of matrigel mixed with Hp_BxPC-3_Exos or PBS were injected into the symmetrical dorsum (Fig. 8A). After 10 days, we removed matrigel and detected the hemoglobin (Hb) concentration and new microvascular density. Exosomes group had higher Hb concentration compared with PBS group (Fig. 8B). Immunohistochemistry results showed that Hp_BxPC-3_Exos increased the density of new microvascular (cd31^+^) (Fig. 8C-D).

### Diagnostic value of miR-30b-5p in PDAC

Because miR-30b-5p was specifically enriched in PDAC, we validated the potential of miR-30b-5p as diagnostic marker for PDAC. Peripheral blood plasma from 24 healthy subjects and 24 PDAC were collected. Some patients with PDAC (15/24) also had portal vein plasma collected during operations (Supplementary Table S3). We simultaneously detected the expression levels of total miR-30b-5p and exosomal miR-30b-5p in plasma, and found that PDAC patients had higher levels of total miR-30b-5p and exosomal miR-30b-5p in peripheral blood plasma than healthy subjects (Fig. 7E, H). There were significant correlations for the level of total miR-30b-5p or exosomal miR-30b-5p between peripheral blood plasma and portal vein plasma (R^2^ = 0.7256, P_value < 0.0001; R^2^ = 0.6291, P_value = 0.0004), and they had similar levels of total miR-30b-5p or exosomal miR-30b-5p (Fig. 7F, I). Receiver operating characteristics (ROC) curve indicated that both total miR-30b-5p and exosomal miR-30b-5p in peripheral blood plasma were eligible for diagnostic markers for PDAC (AUC = 0.9826, 95% CI = 0.9572 - 1; AUC = 0.934, 95% CI = 0.8687 - 0.9994) (Fig. 7G, J).

Taken together, these results suggest that pancreatic cancer cells in hypoxic tumor microenvironment secret large amounts of exosomes, which encapsulate mature miR-30b-5p and transfer them to endothelial cells to suppress the expression of GJA1, and promote tumor angiogenesis (Fig. 8E).

## Discussion

Pancreatic cancer is characterized by the obvious heterogeneity with complex cellular composition^27^. Although conventional bulk-seq helps find aberrantly expressed genes in tumor tissues, it only detects the average gene expression level of the whole tissue, masking the key genes change from minority cell subpopulations. Pancreatic cancer has a low microvascular density, and the transcriptional profile characteristics of endothelial cells are hard to be identified by bulk-seq. Moreover, HUVEC as angiogenesis in vitro could not mimic the real vascular condition in PDAC microenvironment. In this study, we compared the profiles of endothelial cells between PDAC and normal pancreas by scRNA-seq analysis. The result directly reflected the transcriptional profile of endothelial cells in PDAC in situ. We found that DEGs were related to hypoxia and angiogenesis, consistent with the perception that PDAC is in a hypoxic microenvironment^28^, ^29^. In particular, endothelial cells in PDAC expressed HIF-1α and many marker genes involved in the regulation of angiogenesis. Multiple studies have demonstrated that the activation of hypoxia-related signaling pathway was the hallmark of many solid tumors. It contributed to adapt to hypoxia, increase invasion, metastasis and angiogenesis^30^, ^31^. Tumor angiogenesis was regulated by many factors, like VEGF, FGF, TSP-1 secreted by tumor cells and CAFs in PDAC^5^, ^32^. However, the regulation of angiogenesis mediated by exosomes remains largely unknown.

The cell-to-cell communication network mediated by exosomes among tumor cells, PSCs, immune cells has gained wide attention. The relationship between endothelial cells and other cell compositions in PDAC need to be further explored. Guo et al.^33^ reported that pancreatic cancer derived exosomal LncRNA UCA1 promoted angiogenesis through miR-96-5p/AMOTL2 axis, but PBS was used as the control group that could not exclude the protein contamination precipitated during exosomes isolation. Here, we used normal ductal cell (HPNE) as the reference group to observe the effects of normoxic and hypoxic pancreatic cancer cells derived exosomes on angiogenesis. The experimental design was more convincing. In line with the results reported by Guo, this study demonstrated pancreatic cancer cells secreting exosomes under hypoxic condition increased tube formation and endothelial cells migration. In addition, we did not found hypoxic pancreatic cancer cells secreting exosomes promoted vascular permeability and transendothelial cells migration, which may be attributed to limited concentration of exosomes used in these experiments. Further investigation is required to explore the role of exosomes in vascular permeability and tumor metastasis.

Recent studies explored the cell-to-cell communication mediated by exosomes between tumor cells and endothelial cells in different malignancies. Colorectal cancer cell derived exosomes miR-25-3p targeted KLF2 and KLF4 to regulate the expression of VEGFR2, ZO-1, occludin and Claudin5, increase vascular permeability and promote angiogenesis^24^. Hypoxic multiple myeloma cells secreted exosome encapsulated miR-135b to enhance angiogenesis^34^. Metastatic breast cancer cells derived exosomal miR-105 destroyed blood barriers to promote metastasis^23^. In addition, similar to our study, Hsu et al. found hypoxic lung cancer secreted exosomal miR-23a significantly increased tube formation length, endothelial cells migration and vascular permeability compared with normoxic normal epithelial cells and lung cancer cells. Therefore, that tumor cells promote angiogenesis via exosomal miRNAs might be the another hallmark of solid cancers.

MiR-30b-5p exhibited both tumor suppressor and tumor promotion functions in various tumors. A grow body of research reported that miR-30b-5p suppressed malignant behaviors of esophageal squamous cell carcinoma, malignant glioma, liver cancer, renal cell carcinoma^35^-^39^. However, Wu et al.^40^ found that miR-30b-5p promoted the proliferation, migration and invasion of breast cancer cells. Moreover, the diagnostic and prognostic value of miR-30b-5p in peripheral blood and tumor tissues for renal cell carcinoma and breast cancer had been explored^41^-^43^. Our study demonstrated that hypoxic pancreatic cancer cells promoted angiogenesis through transferring exosomal miR-30b-5p to endothelial cells to regulate directly the expression of GJA1. Patients with PDAC had higher expression levels of total miR-30b-5p and exosomal miR-30b-5p in peripheral blood plasma than health subjects. Thus, understanding of the role of exosomal miR-30b-5p in angiogenesis help develop new anti-angiogenesis therapy strategies, and miR-30b-5p has the potential as a diagnostic marker for PDAC.

GJA1 gene is located in 6q22.31, encoding a member of cell junction protein family (Connexin 43). The Connexin 43 builds connections between adjacent cells through mediating the exchanges of small peptide segments, nucleic acids, ions and nutrients^44^. Zhang et al.^45^ reported that Connexin 43 suppressed osteosarcoma cell proliferation by mediating intercellular gap junction communication to increase the expression level of p27. Connexin 43 regulated physiological and pathological processes through transferring drugs and nucleic acids^46^, ^47^. Here, our study found that hypoxic pancreatic cancer derived exosomal miR-30b-5p regulated the expression of GJA1 in endothelial cells to promote angiogenesis. Combined with scRNA-seq results, we hypothesized that exosomal miR-30b-5p/GJA1 pathway might participate in tumor angiogenesis under hypoxic condition in PDAC through regulating gap junction communication. However, it needs to be further investigated. Interestingly, recent studies reported the functions of cell junction proteins in tumor angiogenesis. For example, the dysregulation of tight junction protein (ZO-1) increased vascular permeability to promote metastasis^23^, ^48^. VE-cadherin, occludin and Claudin 5 also played a key role in tumor angiogenesis^22^, ^23^. However, our study did not detect that exosomal miR-30b-5p regulated cell junction proteins above.

Our work initially explored the mechanism of tumor cells derived exosomes promoting angiogenesis in PDAC. But, there are many limitations in this study, such as limited clinical specimens to verify the diagnostic value of exosomal miR-30b-5p for PDAC. Large-scale cohort was needed to explore whether exosomal miR-30b-5p could distinguish PDAC from chronic pancreatitis, benign pancreatic tumor, other malignant diseases. In addition, the relationship between exosomal miR-30b-5p and vascular invasion and distant metastasis and specific mechanism of GJA1 regulating angiogenesis remain to be explored.

In conclusion, our findings shed light on the transcriptional profile characteristics of endothelial cells in PDAC and mechanism by which pancreatic cancer cell derived exosomes regulate tumor angiogenesis, which are helpful for developing new anti-angiogenesis therapy strategies for improving the prognosis of patients with PDAC.

## Methods

### Human samples for scRNA-seq analysis and miR-30b-5p detection

The raw gene-cell matrix was retrieved from GSA database (Genome Sequence Archive) 49 under the access number: CRA001160. A total of 24 untreated PDAC and 11 normal pancreas tissues from Department of General Surgery of Peking Union Medical College Hospital (PUMCH) were enrolled. All paired peripheral blood plasma and portal vein plasma were collected during operation from Department of General Surgery of Peking University First Hospital (PKUFH). Only peripheral blood plasma was collected from patients who did not undergo surgery and healthy subjects. Fresh plasma was preserved in EDTA anticoagulation tube and immediately centrifuged at 1500g at 4°C for 20 minutes, then supernatant was centrifuged at 2500g at 4°C for 15 minutes.

This study was approved by Ethics Committee of Peking University First Hospital (Approval No.JS-1491) and performed in accordance with ethical guidelines (Declaration of Helsinki). Written informed consent was obtained from all participants.

### scRNA-seq data processing, quality control and clustering

The raw gene-cell matrixes from individual specimens were merged and loaded into R package Seurat (v3.2.3). Then, gene-cell matrixes were filtered to remove cells (<200 genes/cell, >10% mitochondria genes, <1000 or >20000 transcripts/cell) and genes (<10 cells/gene). Gene expression levels were normalized (LogNormalize). Total 2000 highly variable genes were acquired and used to conduct PCA reduction dimension. Single cell clustering was performed with Uniform manifold approximation and projection (u-MAP) or t-distributed stochastic neighbor embedding (t-SNE). Marker genes of each cluster matched with signature genes of known cell populations reported in precious literatures and CellMarker database. Violin plot showed the expression levels of selected signature genes in each cluster. Dot plot showed Top 5 marker genes of each cluster.

### Cellular proportion and cell cycling analysis

The proportion of cell subpopulations in PDAC and normal pancreatic specimens was counted. The cell cycling score of each cell was calculated using CellCycleScoring method in the Seurat package, then classified into three statuses including G1, S and G2M. We counted the proportion of cell cycle statuses in PDAC and normal pancreatic specimens, and compared it between tumor cells and normal ductal cells.

### Differential expression genes analysis and annotation

FindMarkers algorithm in Seurat package was applied to identify differential expression genes between two subpopulations (logfc.threshold = 0.5, q-value < 0.05), then volcano plot visualized the result. The clusterProfiler (v3.18.0) package was used to conduct GO and KEGG analysis. The normalized gene-cell matrix was exported to perform genes annotation using GSEA tools (v4.0.1) with default parameters^50^.

### Cell culture under normoxic and hypoxic conditions

Human pancreatic cancer cell lines, including AsPC-1, BxPC-3, MIA PaCa-2, PANC-1, T3M4 and normal pancreatic ductal cell (hTERT-HPNE) were purchased from the American Type Culture Collection (ATCC). Human pancreatic cancer cell line PaTu8988 was from DSMZ. Human umbilical vein endothelial cell (HUVEC) was purchased from the Pharmlab in china. The HEK-293T was generously provided by professor Mao in the department of Biochemistry and Molecular Biology in Peking university. All cell lines were authenticated by short tandem repeats (STR).

The hTERT-HPNE was cultured in in 75% DMEM without glucose (Gibco, USA) and 25% M3 Base (Incell, USA) supplemented with 10 ng/ml human recombinant EGF (CST, USA), 5.5 mM D-glucose (1 g/L), 5% fetal bovine serum (Gibco, USA), 0.75 mg/ml puromycin (sigma, USA). AsPC-1, BxPC-3, T3M4 were cultured in RPMI 1640 (Gibco, USA). MIA PaCa-2, PANC-1, PaTu8988, HEK-293T were cultured in DMEM. HUVEC was cultured in Endothelial Cell Medium (ScienCell, USA) supplemented with 5% fetal bovine serum, endothelial cell growth supplement, 1% penicillin-streptomycin solution (KeyGEN, china). Cells were maintained in cell incubator (ThermoFisher, USA) with 5% CO2 and 20% O2 for normoxic condition and incubator chamber (Billups-rothenberg, USA) flushed with 5% CO2 and 95% N2 for hypoxic condition.

### Western blotting

Cells and exosomes were lysed in lysis buffer (Beyotime, #P0013, China) supplemented with protease inhibitor cocktail (Roche, USA) and PMSF (sigma, USA). Equal amounts of protein were loaded to SDS-PAGE gel, then transferred to PVDF membrane with 0.22 um pore size (Milipore, USA). The PVDF membrane was blocked with skimmed milk, incubated with primary antibody in 4°C overnight, then washed three times with TBS-T. Each PVDF membrane was incubated with secondary antibody in room temperature for 1 hour and added ECL western HRP substrate (Milipore, USA). The following primary antibodies were used: CD9 (SBI, #EXOAB-CD9A-1, 1:1000, USA), CD63 (SBI, #EXOAB-CD63A-1, 1:1000, USA), HSP70 (SBI, #EXOAB-Hsp70A-1, 1:1000, USA), TSG101 (Proteintech, #14497-1-AP, 1:500, USA), Alix (Proteintech, # 12422-1-AP, 1:500, USA), HIF-1α (CST, # 36169S, 1:1000, USA), β-Actin (Proteintech, #66009-1-Ig, 1:5000, USA), GAPDH (Proteintech, #60004-1-Ig, 1:5000, USA), GJA1 (CST, #83649S, 1:1000, USA).

### Exosomes isolation and identification

Multilayer culture disk was used to expand cells in a large-scale. The cell supernatant was harvested when cells were cultured in basic medium supplemented with 10% exosome-depleted fetal bovine serum (100000g, 4°C, 16 hours). Exosomes from cell supernatant were extracted using differential centrifugation method (1500g, 4°C, 20 minutes; 2500g, 4°C, 15 minutes; 10000g, 4°C, 30 minutes; filtered by 0.22 um needle filter; 100000g, 4°C, 70 minutes × 2). Exosomes from EDTA anticoagulation plasma were also extracted using differential centrifugation method.

For transmission electron microscopy (TEM), isolated exosomes were diluted by PBS, stained with 2% uranium dioxide acetate for 1 minute, then transferred to carbon-coated copper grids. Typical characteristics of exosomes in different magnification fields were captured with TEM (Hitachi H-7500). For western blotting, exosomal markers, including CD9, CD63, HSP70, TSG101, Alix, were used to verify exosomal identification compared with parental cells. For nanoparticle tracking analysis (NTA), exosomes diluted 500 times with PBS, then loaded to injection tube with 1 ml syringe. The standard operating procedure was run with default parameters. The size and concentration of exosome were analyzed by NTA (NanoSight NS3000).

### Exosome uptake assay

Exosomes were stained with PKH67 dye (sigma, USA) in room temperature for 2 minutes, then stopped with 1% BSA. Exosomes were stained with PBS as the negative control. The labeled exosomes were centrifuged at 100000g for 70 minutes. The HUVEC was co-incubated with PKH67-exosomes at 37°C for 4 hours, then stained with CM-Dil (KeyGEN, china) and Hoechst33342 (KeyGEN, china). Represent pictures were captured using the confocal microscope (Leica, Germany).

### Tube formation, endothelial cell migration

For tube formation, HUVEC was treated with exosomes (0.1ug/ul) extracted from normoxic and hypoxic MIA PaCa-2 and BxPC-3, normoxic HPNE for 24 hours, then plated on thawed matrigel (Corning, USA) at 4°C in 96 well plate. After 4 hours, five pictures for each well were obtained randomly using inverted fluorescence microscope (Leica, Germany). Total tube formation length was calculated using ImageJ (v1.52v) plug-in for angiogenesis.

For endothelial cell migration, HUVEC was treated with exosomes (0.1ug/ul) and plated on 24-well transwell insert with 8-um pore size, complete medium was added to bottom chamber. After 12 hours of incubation, HUVEC was fixed with 4% paraformaldehyde (Solarbio, China) and stained with 0.1% crystal violet (Solarbio, China). Five pictures for each well were obtained randomly to calculate the number of endothelial cells that migrated to the lower insert membrane.

### Exosomal small RNA-seq

Exosomes from cell supernatant were isolated using differential centrifugation method. Exosomal specific miRNAs and 16S were detected to exclude the contamination of bacterial and mycoplasma. After passing quality control, exosomal small RNAs were extracted and libraries were constructed. The sequencing platform was Illumina HiSeq2500. Sequence align was performed using Silva, GtRNAdb, Rfam, Repbase databases. The rRNA, tRNA, snRNA, snoRNA and duplicate sequence were filtered. The rest of reads were aligned with reference genome (GRCh38) and miRBase. The new miRNAs were predicted using miRDeep2 tool (v2.0.5). The amplification bias was corrected using UMI method. The expression levels of miRNAs were normalized with TPM method. Differential expression miRNAs were identified by edgeR package (v3.32.1). The screening criteria were defined as logfc > 1, P_value < 0.05, TPM > 10.

### Conventional RNA-seq

HUVEC was harvested after exosomes treatment for 24 hours. Total RNA of HUVEC was extracted, then quality control was performed using Agilent2100 (RNA >= 200 ng, 28S/18S >= 1.0, RIN >= 7.0). The cDNA libraries were constructed to perform RNA-seq using DNBSEQ platform (BGI, china). Cleans reads were filtered and aligned with reference genome (GCF_000001405.38_GRCh38.p12). The expression levels of mRNAs were normalized using FPKM method. Differential expression mRNAs were identified by utilizing Dr.Tom online analysis platform (BGI, china). The screening criteria were defined as logfc > 1, P_value < 0.05. Visualization of results, heatmap, upsetplot and venn plot were performed using TBtool software^51^.

### mRNAs, miRNAs isolation and RT-qPCR

Exosomal miRNAs were extracted using miRNeasy Mini Kit (QIAGEN, Germany) according to the manufacturer's instruction. Cel-miR-39-3p was used as the external reference. First-strand cDNAs were synthesized using miDETECT A Track^TM^ method (Ribobio, china). For mRNAs isolation, total RNA was extracted by TRIzol reagent (Invitrogen, USA). First-strand cDNAs were synthesized from the 2 ug total RNA with ReverTra Ace qPCR RT kit (TOYOBO, Japan). Quantitative real-time polymerase chain reaction (RT-qPCR) was performed using SYBR Green Realtime PCR Master Mix (TOYOBO, Japan). The relative expression level was calculated using 2^-△△CT^ method. The following primers were used: Pri-mir-30b-F: TTGTTTGGTAGTTGGGGTCGG; Pri-mir-30b-R: TCTGATGTCAAAGCCCATGCT; Pre-mir-30b-F: GCCGAGACCAAGTTTCAGTTC; miR-30b-5p-F: GCCGAGTGTAAACATCCTAC; Common-miR-F: CAGTGCGTGTCGTGGAGT; CLDN5-F: GAGCAGCCCCTGTGAAGATT; CLDN5-R: GTCTCTGGCAAAAAGCGGTG; OCLN-F: CCAATGTCGAGGAGTGGGTT; OCLN-R: TGCCATGGGACTGTCAACTC; GJA1-F: CAGCCACTAGCCATTGTGGA; GJA1-R: CCATACACCCCCAGTGAACC; TJP1-F: ATGGAGGAAACAGCTATATGGGA; TJP1-R: CCAAATCCAAATCCAGGAGCC; GAPDH-F: GTATTGGGCGCCTGGTCACC; GAPDH-R: CGCTCCTGGAAGATGGTGATGG.

### Gain/loss of function

For miRNAs, miRNA mimics and inhibitors were transferred to cells using Lipo3000 (Invitrogen, USA). For genes, plasmids for knockdown and overexpression were designed using lentivirus vectors by the Syngentech company in china. The following plasmids were used: GJA1-OE: pLV-hef1a-mNeongreen-P2A-Puro-WPRE-CMV-GJA1(Human, NM_000165)-3Xflag; Vector-OE: pLV-hef1a-mNeongreen-P2A-Puro-WPRE-CMV-Vector.

### Exosomal miRNAs protection assay

Exosomes were extracted using differential centrifugation method and treated with or without 50U RNase If (New England Lab, USA). Then, exosomes were incubated with 0.1% Triton X-100 (sigma, USA) at 37°C for 30 minutes. Enzyme inactivation at 75°C for 5 minutes was conducted. The expression of miR-30b-5p was detected.

### Dual luciferase report assay

The 3' UTR region of GJA1 was inserted into the dual luciferase report vector pMIR-REPORT-GJA1-3' UTR (WT/MT). The 293T was transferred with firefly luciferase vector, renilla luciferase vector and miR-30b-5p mimic using Lipo3000. Relative fluorescence intensity was detected using the Dual-Luciferase Reporter Assay System (Promega, USA) by the chemiluminescence instrument (Centro LB960).

### Immunohistochemistry

Immunohistochemistry (IHC) was performed on freshly prepared 4% paraformaldehyde fixed paraffin-embedded tissue sections. Sections were deparaffinized in xylene, rehydrated in decreasing concentration of ethanol, then washed with PBS. Sections were added 3% hydrogen peroxide to remove endogenous peroxidase activity. Put the slides in a slide rack and placed it in 0.01 M citrate buffer, then kept it boiling for 10 minutes and cooled down for at least 10-20 minutes at room temperature for epitope retrieval. Sections were blocked with 10% goat serum at room temperature for 60 minutes and incubated with primary antibody for cd31 (CST, #77699S, 1:200, USA) at 4°C overnight in a humidified chamber. Finally, each section was treated with HRP conjugated secondary antibody at room temperature for 60 minutes. Fresh diaminobenzidine (DAB) was added to each section. The cell nucleus was counterstained with Mayer's hematoxylin (Biodee, china). Five pictures of each section were obtained randomly to calculate the expression level of cd31.

### Matrigel plug assay

Equal amounts of exosomes (40 ug/100 ul) were mixed with thawed matrigel (400 ul), then injected subcutaneously into symmetrical dorsum in C57BL6/J (male, 8-week-old, n = 4). After 10 days, mice were euthanized, and matrigel plugs were harvested to perform IHC, hemoglobin (Hb) detection with Van KampenZijlstra reagent (Xin Fan, China).

### Statistical analysis

Each experiment was conducted in triplicate wells in triplicate experiments independently. For continuous variable subjected to normal distribution, the independent-samples t test was performed to compare means between two groups, and one-way ANOVA was conducted to compare means among many groups. For categorical variable, the chi-square test or rank sum test was used. Correlation analysis was conducted using Pearson/Spearman test. Non-parametric test was used for data that were not subjected to normal distribution. All statistical analyses were conducted using SPSS version 22.0 software (SPSS22, Chicago, USA). Statistical significance was defined as *p < 0.05, **p < 0.01, ***p < 0.001.

## Supplementary Material

Supplementary figures and tables.
