# Supplementary material for: Hypoxic pancreatic cancer derived exosomal miR-30b-5p promotes tumor angiogenesis by inhibiting GJA1 expression
Source: Int J Biol Sci. 2022 Jan 1;18(3):1220–37. doi: 10.7150/ijbs.67675 (PMC8771853; doi:10.7150/ijbs.67675)
Supplement: Supplementary file 1 — Supplementary figures and tables. [file ijbsv18p1220s1.pdf]

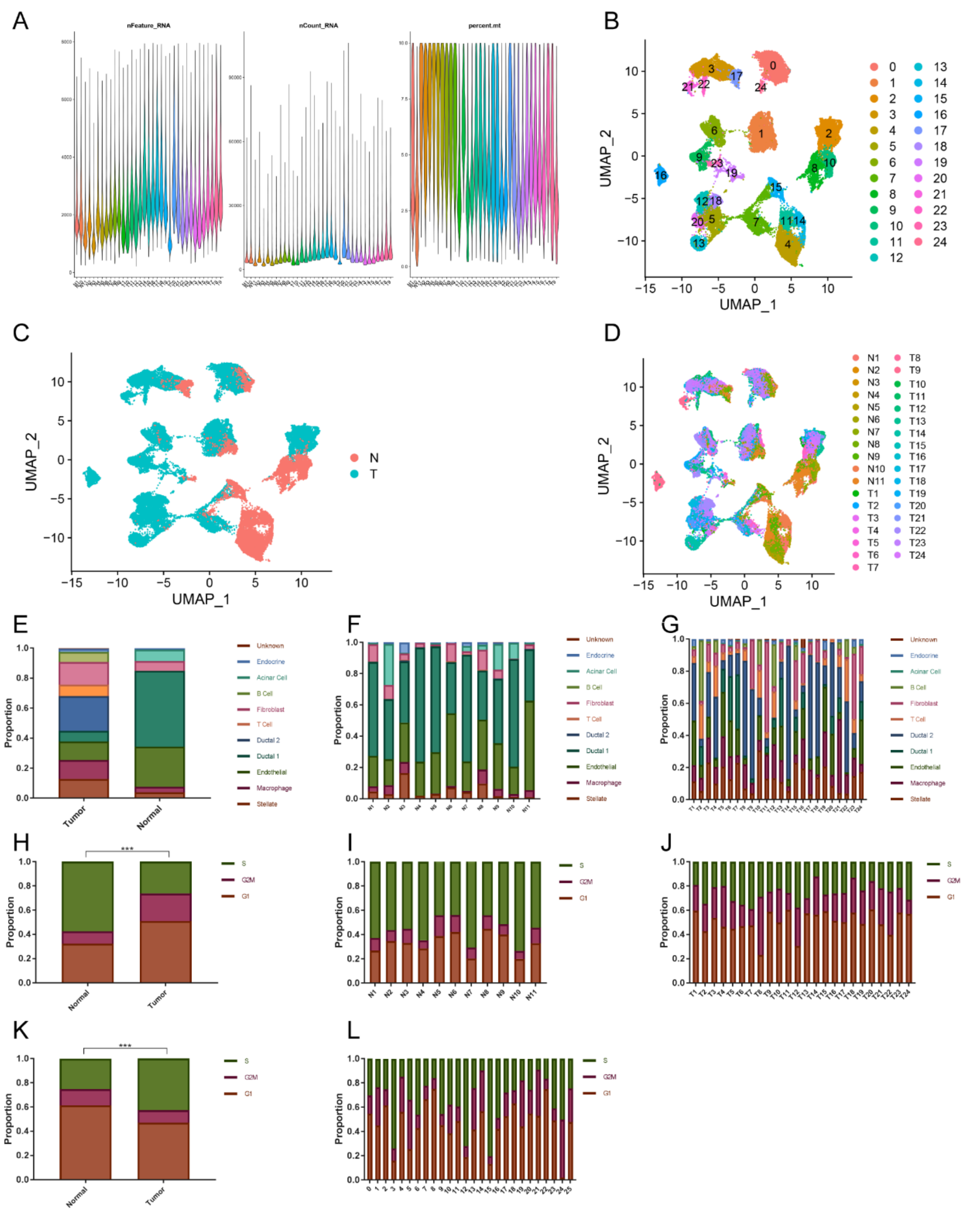

Fig.S1 Cell composition and cell cycling analysis. (A) Quality control of scRNA-seq analysis. (B-D) u-MAP plot showing original clusters (B), clusters from PDAC and normal pancreatic specimens (C), clusters from various specimens (D). (E-G) The proportion of cell populations for PDAC vs normal pancreatic specimens (E), normal specimens (F), PDAC specimens (G). (H-J) The proportion of cell cycling state of cell populations for PDAC vs normal pancreatic specimens (H), normal specimens (I), PDAC specimens (J). (K) The proportion of cell cycling state of endothelial cells in PDAC and normal pancreatic specimens. (L) The proportion of cell cycling state of cell

populations for original clusters.

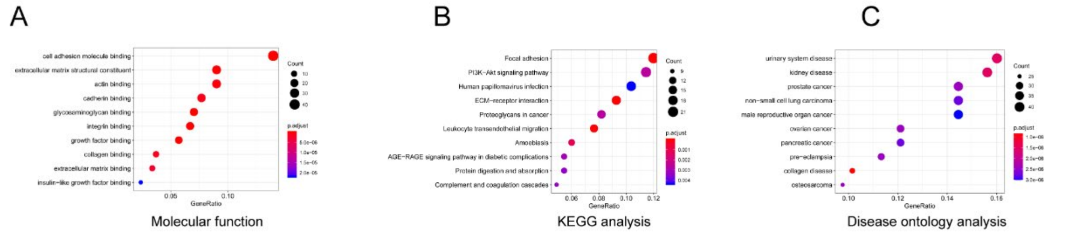

D

NAME	SIZE	ES	NES	FDR q-val	FWER p-val
HALLMARK_COAGULATION	116	0.589	2.556	0	0
HALLMARK_IL2_STAT5_SIGNALING	189	0.544	2.511	0	0
HALLMARK_INFLAMMATORY_RESPONSE	183	0.546	2.458	0	0
HALLMARK_COMPLEMENT	187	0.529	2.432	0	0
HALLMARK_HYPOXIA	182	0.527	2.397	0	0
HALLMARK_ANGIOGENESIS	34	0.694	2.382	0	0
HALLMARK_PI3K_AKT_MTOR_SIGNALING	98	0.529	2.273	0	0
HALLMARK_GLYCOLYSIS	185	0.482	2.241	0	0
HALLMARK_WNT_BETA_CATENIN_SIGNALING	40	0.518	1.905	0.000425	0.007

E

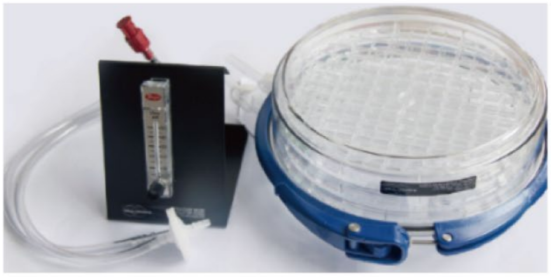

F

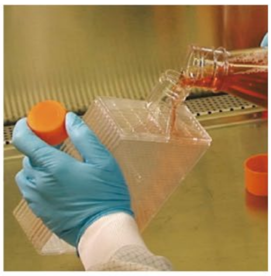

Fig.S2 Endothelial cells in PDAC were related angiogenesis and hypoxia. (A-C) Gene ontology analysis showing molecular function (A), KEGG pathway (B), disease (C) terms of upregulated genes in Endothelial 1, colorful dots represented the level of statistical significance. (D) The top hallmark terms in GSEA analysis. (E) Hypoxic incubator chamber. (F) Multilayer culture flask was used to expand cells in a large-scale.

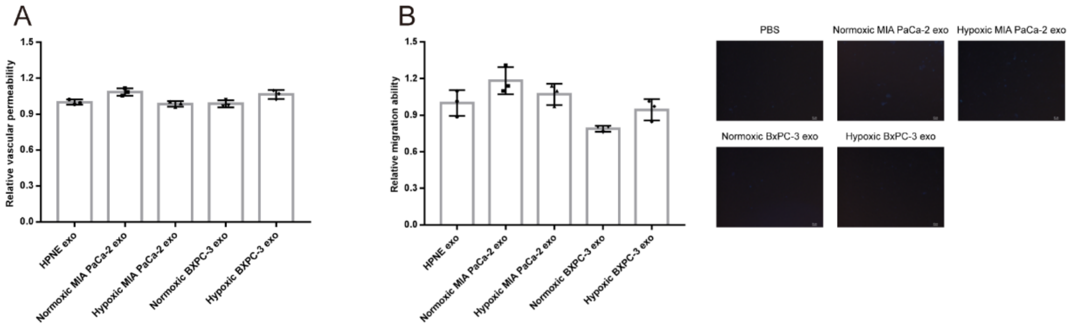

C

Samples	Total Reads	Mapped Reads	Q30(%)	Known miRNAs	Novel miRNAs	Total miRNAs
HPNE exo	27495472	6714811	96.79	815	25	840
Normoxic BxPC-3 exo	15022843	2005010	96.58	797	22	819
Hypoxic BxPC-3 exo	8208967	829732	97.27	643	18	661

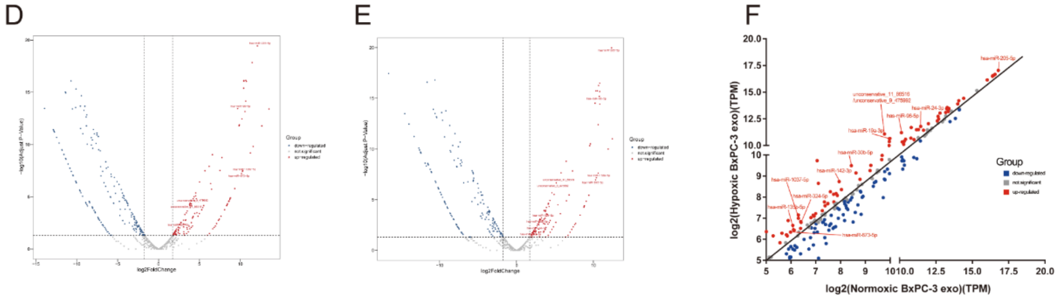

G

Samples	Total Clean Reads (M)	Mapped Reads (%)	Q30(%)	Clean Reads Ratio (%)
WT	45.19	75.7	91.9	95.49
HPNE exo	45.5	74.96	92.16	96.14
Normoxic BxPC-3 exo	45.17	76.75	91.97	95.59
Hypoxic BxPC-3 exo	45.31	75.54	91.72	95.73

Fig.S3 Exosomal small RNA-seq and conventional RNA-seq of HUVEC after exosomes treatment. (A) HUVEC permeability was tested using FITC-dextran with 70000 MW. (B) The transendothelial cells ability of tumor cells labeled with Hoechst33342 was tested. Blue dot represented the nucle of tumor cells. (C) The basic results of exosomal small RNA-seq. (D-E) Volcano plot showing differential expression miRNAs between No_HPNE_Exos and No_BxPC-3_Exos (D), No_HPNE_Exos and Hp_BxPC-3_Exos (E). (F) Dot plot showing differential expression miRNAs between No_BxPC-3_Exos and Hp_BxPC-3_Exos. (G) The basic results of RNA-seq of HUVEC.

Table S1. Clinicopathologic characteristics of specimens by scRNA-seq analysis.

D	Diagnosis	Sex	Age (Y)	CA199(U/ml)	Diabetes	Procedure	Location	Diameter (mm)	TNM	AJCC stage
T1	moderately-poorly differentiated PDAC	M	64	86.0	N	LDP	body	26	T4N2M0	III
T2	well differentiated PDAC	M	52	46.3	N	PD	head	20	T1cN1M0	IIB
T3	moderately-poorly differentiated PDAC	F	58	49.2	Y	PD	uncinate process	22	T2N0M0	IB
T4	moderately differentiated PDAC	F	72	40.4	Y	LDP	body	14	T1cN1M0	IIB
T5	well-moderately differentiated PDAC	F	65	37.0	Y	PD	uncinate process	29	T2N0M0	IB
T6	moderately-poorly differentiated PDAC	M	64	155.1	N	ODP	tail	91	T3N0M0	IIA
T7	moderately differentiated PDAC	M	70	<0.6	Y	ODP	body	80	T3N1M0	IIB
T8	moderately-poorly differentiated PDAC	F	66	82.5	N	PD	uncinate process	17	T1cN2M0	III
T9	moderately-poorly differentiated PDAC	M	36	11.2	N	PD	head	26	T2N0M0	IIA
T10	poorly differentiated PDAC	M	61	972.8	Y	PD	uncinate process	40	T2N1M0	IB
T11	moderately-poorly differentiated PDAC	M	51	211.1	N	ODP	body and tail	76	T3N1M0	IIB
T12	poorly differentiated PDAC	M	54	146.1	N	PD	uncinate process	50	T3N2M0	III
T13	moderately-poorly differentiated PDAC	F	58	21.9	Y	PD	head	30	T2N1M0	IIB
T14	well differentiated PDAC	F	67	77	Y	PD	head	33	T2N1M0	IIB
T15	well differentiated PDAC	F	54	18.4	N	LPD	head	23	T2N1M0	IIB
T16	poorly differentiated PDAC	F	56	42.9	N	LDP	body	30	T2N1M0	IIB
T17	moderately differentiated PDAC	F	71	209.3	N	LDP	body and tail	30	T2N0M0	IB

T18	moderately-poorly differentiated PDAC	F	68	112.3	Y	ODP	body	28	T2N0M0	IB
T19	well-moderately differentiated PDAC	F	59	93.9	N	LPD	head	35	T2N0M0	IB
T20	moderately differentiated PDAC	M	59	2.2	N	PD	head	43	T3N1M0	IIB
T21	moderately-poorly differentiated PDAC	M	59	528.6	Y	LPD	head	35	T2N0M0	IB
T22	moderately differentiated PDAC	F	67	234.5	N	ODP	body	27	T2N0M0	IB
T23	moderately-poorly differentiated PDAC	M	54	312.2	Y	PD	head	27	T2N1M0	IIB
T24	moderately differentiated PDAC	F	44	14.4	N	PD	head	20	T1cN0M0	IB
N1	normal pancreas/mucinous cystic neoplasia	F	64	7.5	N	ODP	tail	50	NA	NA
N2	normal pancreas/small intestine papillary adenocarcinoma	M	55	171.2	N	PPPD	descending duodenum	11	NA	NA
N3	normal pancreas/duodenal intraepithelial neoplasia	M	50	6.4	N	PD	descending duodenum	20	NA	NA
N4	normal pancreas/pancratic neuroendocrine tumor	M	53	4.5	N	LDP	body and tail	40	NA	NA
N5	normal pancreas/serous cystic neoplasia	F	52	9.0	N	LDP	body and tail	24	NA	NA
N6	normal pancreas/solid pseudopapillary tumor	F	31	29.5	N	ODP	body	22	NA	NA
N7	normal pancreas/mucinous cystic neoplasia	F	42	12.7	N	LDP	tail	94	NA	NA
N8	normal pancreas/solid pseudopapillary tumor	M	41	6.0	N	LDP	body and tail	76	NA	NA
N9	normal pancreas/pancratic neuroendocrine tumor	M	34	23.8	N	LDP	tail	22	NA	NA
N10	normal pancreas/choledochal neuroendocrine tumors	F	65	193.3	N	PD	common bile duct	NA	T3N0M0	IIA
N11	normal pancreas/solid pseudopapillary tumor	F	30	NA	N	LDP	body	33	NA	NA

ODP, Open distal pancreatectomy; LDP, Laparoscopic distal pancreatectomy; PD, Pancreatoduodenectomy; LPD, Laparoscopic pancreatoduodenectomy; PPPD, Pylorus preserved pancreatoduodenectomy

Table S2. Signature genes for clusters identification.

Subpopulations	Signature genes
Ductal Cell	AMBP, CFTR, MMP7, SOX9, LCN2, KRT18, KRT8, TFF2, FXYD2, TSPAN8
Macrophage	AIF1, CD68
B Cell	MS4A1, VPREB3, CD79A, CD79B
Endothelial Cell	CDH5, RAMP2, PLVAP, VWF
Stellate Cell	RGS5, NDUFA4L2
T Cell	CD3D, CD3E, CD2
Fibroblast	LUM, COL1A1, SFRP2, COL3A1, DCN
Acinar	PRSS1, CTRB2, REG1A, REG1B
Endocrine	CHGB, CHGA, PCSK1N, INS

Table S3. Clinicopathologic characteristics of specimens for miR-30b-5p detection.

ID	Diagnosis	Sex	Age (Y)	BMI	CA199 (U/ml)	TNM	AJCC stage	Diabetes
PKUFH-1	well differentiated PDAC	F	61	23.69	411.2	T2N2M0	III	N
PKUFH-2	moderately-poorly differentiated PDAC	M	63	22.83	>1000	T2N2M1	IV	N
PKUFH-3	moderately differentiated PDAC	F	65	18.38	302.1	T2N1M0	IIB	N
PKUFH-4	moderately-poorly differentiated PDAC	M	65	23.7	>1000	T1N2M0	III	N
PKUFH-5	moderately-poorly differentiated PDAC	M	54	29	277.3	T3N2M0	III	N
PKUFH-6	moderately differentiated PDAC	F	70	24.89	>1000	T2N0M0	IB	N
PKUFH-7	poorly differentiated PDAC	F	84	27.3	249.2	T2N1M0	IIB	N
PKUFH-8	moderately differentiated PDAC	M	60	25.73	472.2	T2N1M0	IIB	N
PKUFH-9	moderately differentiated PDAC	F	67	27.16	108.4	T2N2M0	III	Y
PKUFH-10	poorly differentiated PDAC	M	61	24.5	203.5	T3N1M0	IIB	Y
PKUFH-11	moderately-poorly differentiated PDAC	M	59	24.9	28.38	T4N2M0	III	N
PKUFH-12	moderately differentiated PDAC	M	70	23.16	432.4	T2N0M0	IB	Y
PKUFH-13	poorly differentiated PDAC	F	89	21.27	213.9	T2N0M0	IB	Y
PKUFH-14	moderately-poorly differentiated PDAC	F	62	25.17	>1000	T3N1M0	IIB	Y
PKUFH-15	moderately-poorly differentiated PDAC	M	76	22.49	51.43	T1N2M0	III	N
PKUFH-16	moderately differentiated PDAC	M	73	24.78	372.4	T2N1M0	IIB	N
PKUFH-17	moderately differentiated PDAC	F	59	24.78	115.4	T2N2M0	III	Y
PKUFH-18	poorly differentiated PDAC	M	66	19.98	347.5	T3N1M0	IIB	Y
PKUFH-19	well differentiated PDAC	F	68	20.14	58.78	T3N2M0	III	N
PKUFH-20	moderately differentiated PDAC	F	75	22.14	>1000	T2N1M0	IIB	N
PKUFH-21	poorly differentiated PDAC	F	81	26.45	347.9	T2N1M0	IIB	Y
PKUFH-22	poorly differentiated PDAC	F	62	23.67	>1000	T1N2M0	III	Y
PKUFH-23	moderately-poorly differentiated PDAC	M	76	24.44	179.3	T2N1M0	IIB	Y
PKUFH-24	moderately differentiated PDAC	M	71	27.69	679.3	T1N2M0	III	N
